# Association Between Low T3 Syndrome and Poor Prognosis in Adult Patients With Acute Myocarditis

**DOI:** 10.3389/fendo.2021.571765

**Published:** 2021-03-08

**Authors:** Yan Zhao, Wenyao Wang, Kuo Zhang, Yi-Da Tang

**Affiliations:** ^1^Department of Cardiology, State Key Laboratory of Cardiovascular Disease, Fuwai Hospital, National Center for Cardiovascular Diseases, Chinese Academy of Medical Sciences and Peking Union Medical College, Beijing, China; ^2^Department of Cardiology and Institute of Vascular Medicine, Peking University Third Hospital, Key Laboratory of Molecular Cardiovascular Science, Ministry of Education, Beijing, China

**Keywords:** low T3 syndrome, adult, acute myocarditis, prognosis, predictor

## Abstract

**Background:**

This study aims to investigate the role of free triiodothyronine (fT3) in predicting poor prognosis of adult patients with acute myocarditis.

**Methods:**

A total of 173 consecutive adult patients with acute myocarditis completed thyroid function evaluations. They were divided into two groups according to fT3 levels: low fT3 group (n = 54, fT3 < 3.54 pmol/liter) and normal fT3 group (n = 119, fT3 ≥ 3.54 pmol/liter). The primary endpoint was major adverse cardiac events (MACE).

**Results:**

During the 3.5 ± 2.8 years follow-up, the rate of MACE was 29.6% versus 3.5% in low fT3 group versus normal fT3 group, respectively (*P* < 0.0001). Long-term at 8 years MACE-free survival were lower in low fT3 group versus normal fT3 group (52.9% versus 92.3%, log-rank *P* < 0.0001), respectively. Univariate Cox analysis showed that left ventricular ejection fraction (LVEF) < 50% [hazard ratio (HR) 10.231, 95% confidence interval (CI): 3.418–30.624, *P* < 0.0001) and low fT3 level (HR 0.360, 95% CI: 0.223–0.582, *P* < 0.0001) were strongest two predictors of MACE. After adjustment for traditional risk predictors, the prognostic value of fT3 status was still significant (HR 0.540, 95% CI: 0.316–0.922, *P* = 0.024). Compared with normal fT3 group, those in low fT3 group were at a much higher risk of MACE (HR 5.074, 95% CI: 1.518–16.964, *P* = 0.008).

**Conclusions:**

Low T3 syndrome was a strong predictor of poor prognosis in adult patients with acute myocarditis. These findings suggest that fT3 level could serve as a biomarker for risk stratification in acute myocarditis patients.

## Introduction

The cardiovascular system is a major target on which thyroid hormone act. The heart relies mainly on the biologically active hormone triiodothyronine (T3) (1). Clinical and experimental evidence has shown that T3 plays an important role in maintaining cardiovascular homeostasis and low T3 status can adversely influence cardiovascular outcomes (2, 3). A combination of low serum T3 level and thyroid-stimulating hormone (TSH) within normal range or slightly decreased is called low T3 syndrome, which has been reported in most critically ill patients (4). Accumulating evidence suggests that low T3 status was a strong predictor of death in cardiac patients (5, 6).

Acute myocarditis is an inflammatory disease of myocardium with variable clinical presentations and prognosis according to distinct etiology (7, 8). Cohort studies showed that patients presenting with complicated myocarditis might progress quickly and suffered a greater death risk than those with uncomplicated myocarditis (9, 10). Therefore, it is crucial to identify high risk patients early to provide active treatment and improve patient prognosis. Although endomyocardial biopsy (EMB) remains to be the diagnostic gold standard and outcome predictor for myocarditis, it is not used routinely.

Previous studies indicated that prolonged QRS or QTc interval and decreased LVEF were established predictors associated with poor prognosis in patients with myocarditis (11–13). However, there are limited data focused on the thyroid hormone levels and the prognosis of patients with acute myocarditis. As a biomarker of adverse outcome in cardiac patients, low T3 syndrome might be a promising predictor for risk stratification of acute myocarditis.

Based on the aforementioned information, we hypothesized that low T3 syndrome could serve as an independent predictor of poor prognosis in patients with acute myocarditis. We tested the hypothesis with complete information of thyroid profile from a cohort of 173 patients with acute myocarditis.

## Methods

### Ethics Statement

The study was in accordance with the ethical guidelines of the Declaration of Helsinki and China’s regulations and guidelines on good clinical practice and was approved by the ethics committees of Fuwai Hospital.

### Study Population and Participants

**Figure 1:**
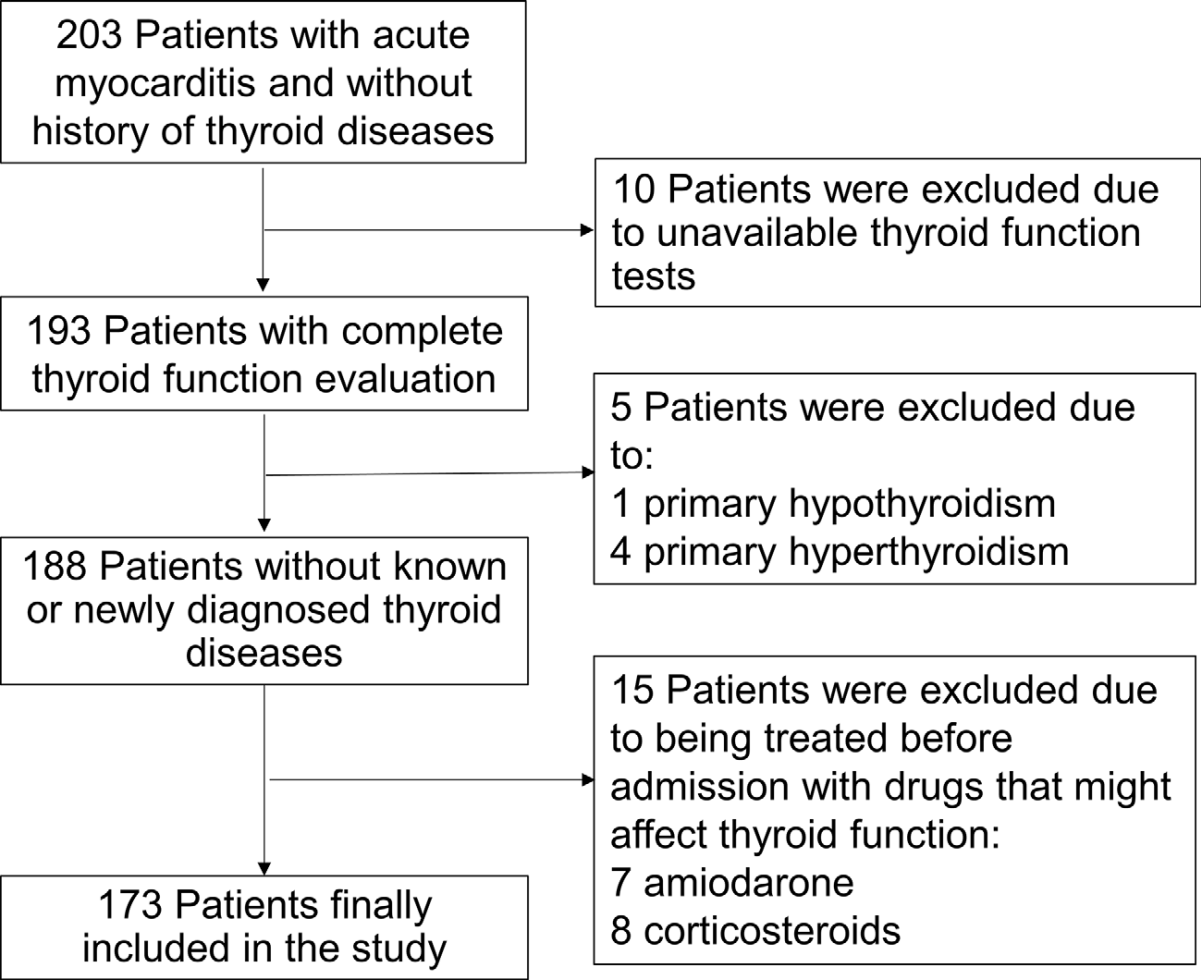
The flow chart of the enrollment of 173 adult patients with acute myocarditis from the overall population with acute myocarditis. 15 patients who had been treated before admission with drugs that might affect thyroid function were excluded.

All patients in this study were evaluated at Fuwai Hospital (National Center of Cardiovascular Diseases, Beijing, China). 203 patients (age ≥13 years) were diagnosed acute myocarditis between September 2009 to February 2019. Clinical information was collected, including medical history, vital signs, laboratory tests and treatments. All patients had no history of thyroid diseases. 10 Patients without available thyroid hormone tests were excluded from the analysis. 5 patients were also excluded from the study because of overt primary hypothyroidism (thyroid-stimulating hormone [TSH] level >18μIU/mL and free T4 [fT4]<11.57 pmol/liter) in 1 patient and hyperthyroidism (free T3 [fT3]>6.47 pmol/liter or fT4>22.88 pmol/liter, with TSH levels <0.02μIU/mL) in 4 patients by thyroid hormone evaluation after admission. 15 Patients who had been treated before admission with drugs that might affect thyroid function, including amiodarone, corticosteroids or antithyroid drugs, were excluded. No patients were on therapy with other drugs that might interfere with TSH and/or thyroid hormone levels, including thyroxine and liothyronine. All patients had no history of hypothalamic-pituitary disorder, autoimmune disease, sarcoidosis, and amyloidosis. The diagnosis of acute myocarditis was based on the European Society of Cardiology expert consensus on diagnosis and management of myocarditis (8): 1) Clinical presentation (new onset within 3 months): acute chest pain, dyspnea, fatigue, palpitation, syncope, heart failure signs and/or aborted sudden cardiac death, unexplained cardiogenic shock; 2) electrocardiography (ECG)/Holter/stress test features; 3) Myocardium injured markers: elevated troponin I/T; 4) Functional and structural abnormalities on echocardiography; 5) Edema and/or late gadolinium enhancement on cardiovascular magnetic resonance imaging, and in the absence of coronary stenosis ≥50%, valvular heart disease, hypertensive heart disease or cardiomyopathy. Thus, 173 patients were retrospectively included in the analysis. According to fT3 levels, patients were divided into two subgroups: low fT3 group (n = 54), patients with fT3 level below the lower limit of the reference interval (fT3<3.54 pmol/liter), and normal fT3 group (n = 119), patients with normal fT3 (≥3.54 pmol/liter). The flow chart of patient enrollment is shown in **Figure 1**.

During hospitalization, all patients were managed according to the recommended treatment for myocarditis (8). Stable patients with injured left ventricular function received evidence-based heart failure treatment. Patients with severe or cardiogenic shock were treated inotropes and mechanic circulatory support (MCS). MCS consisted of an intra-aortic balloon pump (IABP) alone or in combination with venous-arterial extracorporeal membrane oxygenation (va-ECMO).

### Thyroid Function Tests

The thyroid function measurement was evaluated in all patients from 2 to 5 days after the admission. Twelve-hour fasting blood samples were drawn and serum fT3, fT4, TT3, TT4 and TSH were measured by using radioimmunoassay (Immulite 2000; Siemens, Germany) in the Nuclear Medicine Department of Fuwai Hospital. The reference ranges for our laboratory are as follows: total T3 (TT3) 0.92 to 2.79nmol/liter, total T4 (TT4) 57.92 to 140.28nmol/liter, fT3 3.54 to 6.47 pmol/liter, fT4 11.57 to 22.88 pmol/liter, and TSH 0.55 to 4.78μIU/mL. Low T3 syndrome was defined as a combination of low serum T3 and slightly low to normal serum TSH concentration.

### Follow-Up and Endpoints

Follow-up data were obtained from one of the following three sources: interviewing the patient by phone with trained doctor, reviewing patient’s hospital records, examining the patient in the outpatient of our hospital. Major adverse cardiac events (MACE) were defined as:1) all-cause death; 2) heart transplantation; 3) heart failure decompensation requiring hospitalization; 4) documented sustained ventricular arrhythmia (>30s).

### Statistical Analysis

Continuous variables are reported as mean ± SD. Comparison between groups were performed for continuous variables using Student’s *t* test and for categorical variables using *χ^2^* test. Univariate Cox proportional hazards analysis was used to determine which variable might have predicted MACE. To adjust for other risk factors, multivariate Cox analysis was performed with all the variable found to be significant at the univariate analysis as well as traditional risk predictors (age, QRS duration>120ms, QTc interval>440ms and LVEF<50%) entering in a single step. Survival curves of patients grouped by fT3 levels, estimating 30-day and long-term MACE, were calculated by the Kaplan-Meier method and compared with the log-rank test, respectively. The receiver operating characteristic (ROC) curve analysis and the area under the ROC curve (AUC) were used to quantify the ability of the selected risk factors for predicting MACE, where a value of 1.0 represents perfect ability and a value of 0.5 indicates no ability. All tests were 2-sided, and *P* < 0.05 was considered statistically significant. Analyses were performed with SPSS statistics software version 17.0 and the Kaplan-Meier curve were made with GraphPad Prism software version 5.

## Results

### Patients Population and Clinical Presentation

The baseline clinical characteristics of the study population are shown in **Table 1**. Patients were relatively young (mean age 30 years) and 72% were male. There were 54 patients (31% of total population) in low fT3 group and 119 patients in normal fT3 group. The proportion of female patients was larger (44.4% versus 21%, *P* = 0.002), while dyspnea (64.8% versus 28.6%, *P* < 0.0001) and syncope (16.7% versus 4.2%, *P* = 0.012) was more frequent in low fT3 group than in normal fT3 group. The proportion of patients with hypertension was higher in low fT3 group, while no significant differences were found regarding diabetes and dyslipidemia. Significantly lower systolic blood pressure (101.9 ± 12.3 versus 114.5 ± 15.1mmHg, *P* < 0.0001), higher heart rate (91.4 ± 19.5 versus 79.4 ± 16.2 beats/minute, *P* < 0.0001), prolonged QTc interval (463.7 ± 45.2 versus 431.7 ± 43.1ms, *P* < 0.0001), higher percentage of sustained ventricular arrythmias (18.5% versus 0, *P* < 0.0001) as well as complete atrioventricular block (22.2% versus 6.7%, *P* = 0.003) were presented in low fT3 group than in normal fT3 group. Increased inflammatory biomarker such as C response protein (CRP) was more prevalent (85.2% versus 59.8%, *P* = 0.001) in low fT3 group. LVEF was significantly lower (46.5 ± 13.0% versus 58.9 ± 11.2%, *P* < 0.0001) in low fT3 group than in normal fT3 group. In terms of management, patients in low fT3 group needed more invasive life support treatments (18.5% versus 1.7%, *P* < 0.0001).

**Table 1:** Clinical Characteristics of 173 Patients with Acute Myocarditis.

	Total (n = 173)	Low fT3 group (n = 54, 31%)	Normal fT3 group (n = 119, 69%)	*t/z/χ^2^*	*P* Value
Demographics					
Age, y	30 ± 13	36 ± 14	28 ± 11	-3.447	0.001
Male, n (%)	124 (71.7)	30 (55.6)	94 (79.0)	10.049	0.002
BMI, kg/m^2^	23.58 ± 4.30	23.10 ± 4.98	23.78 ± 3.97	0.941	0.348
Comorbidities					
Hypertension, n (%)	11 (6.4)	8 (14.8)	3 (2.5)	9.429	0.004
Diabetes mellitus, n (%)	6 (3.5)	3 (5.6)	3 (2.5)	1.022	0.378
Dyslipidemia, n (%)	13 (7.5)	6 (11.1)	7 (5.9)	1.461	0.230
Clinical presentation, n (%)					
Chest pain	70 (40.5)	17 (31.5)	53 (44.5)	2.628	0.105
Dyspnea	69 (39.9)	35 (64.8)	34 (28.6)	20.350	<0.0001
Syncope	14 (8.1)	9 (16.7)	5 (4.2)	7.760	0.012
Vital signs at admission					
SBP (mmHg)	110.5 ± 15.4	101.9 ± 12.3	114.5 ± 15.1	5.379	<0.0001
DBP (mmHg)	66.7 ± 10.0	65.5 ± 9.9	67.2 ± 10.1	1.002	0.318
HR (beats/min)	83.1 ± 18.1	91.4 ± 19.5	79.4 ± 16.2	-3.925	<0.0001
ECG at admission					
Normal, n (%)	55 (31.8)	6 (11.1)	49 (41.2)	15.4484	<0.0001
QRS interval, ms	99.1 ± 28.0	105.8 ± 33.3	96.1 ± 24.9	-1.891	0.062
QTc interval, ms	441.6 ± 46.1	463.7 ± 45.2	431.7 ± 43.1	-4.447	<0.0001
QRS interval >120ms, n (%)	27 (15.6)	13 (24.1)	14 (11.8)	4.273	0.039
QTc interval >440ms, n (%)	80 (46.2)	38 (70.4)	42 (35.3)	18.384	<0.0001
Arrhythmia, n (%)					
Supraventricular tachycardia	11 (6.4)	6 (11.1)	5 (4.2)	2.978	0.100
Sustained VT/VF	10 (5.8)	10 (18.5)	0	23.389	<0.0001
complete AVB	20 (11.6)	12 (22.2)	8 (6.7)	8.728	0.003
Bundle-branch block	22 (12.7)	10 (18.5)	12 (10.1)	2.381	0.123
Laboratory tests at admission					
Increased CRP, n (%)	116 (67.8)	46 (85.2)	70 (59.8)	10.887	0.001
TT3, nmol/liter	1.40 ± 0.47	0.90 ± 0.38	1.62 ± 0.31	13.081	<0.0001
TT4, nmol/liter	98.68 ± 24.19	83.62 ± 22.99	105.51 ± 21.56	6.061	<0.0001
fT3, pmol/liter	4.10 ± 1.13	2.77 ± 0.49	4.71 ± 0.75	17.407	<0.0001
fT4, pmol/liter	15.75 ± 3.22	13.90 ± 2.87	16.59 ± 3.02	5.522	<0.0001
TSH, μIU/ml	1.84 ± 1.62	1.08 ± 1.23	2.19 ± 1.66	4.377	<0.0001
Echocardiography at admission, n (%)					
LVEDD, mm	49.4 ± 6.9	50.3 ± 7.7	49.0 ± 6.4	-1.177	0.241
LVEF, %	55.0 ± 13.1	46.5 ± 13.0	58.9 ± 11.2	6.088	<0.0001
LVEF <50%, n (%)	50 (28.9)	31 (57.4)	19 (16.0)	31.044	<0.0001
Coronary angiography or CT angiography performed, n (%)	128 (74.0)	36 (66.7)	92 (77.3)	2.187	0.139
No evidence of CAD, n (%)	128 (100)	36 (100)	92 (100)	–	–
Medications					
β-Blockers, n (%)	125 (72.3)	38 (70.4)	87 (73.1)	0.139	0.709
ACE-I or ARB, n (%)	79 (45.7)	18 (33.3)	61 (51.3)	4.811	0.028
Aldosterone antagonists, n (%)	40 (23.1)	20 (37.0)	20 (16.8)	8.552	0.003
Life support treatment					
MCS, n (%)	12 (6.9)	10 (18.5)	2 (1.7)	16.314	<0.0001
Ventilator, n (%)	7 (4.0)	6 (11.1)	1 (0.8)	10.092	0.004
Temporary pacing, n (%)	15 (8.7)	10 (18.5)	5 (4.2)	9.615	0.006

BMI, body mass index; SBP, systolic blood pressure; DBP, diastolic blood pressure; ECG, electrocardiogram; VT, ventricular tachycardia; VF, ventricular fibrillation; AVB, atrioventricular block; CRP, C-reactive protein; TT3, total triiodothyronine; TT4, total thyroxine; fT3, free triiodothyronine; fT4, free thyroxine; TSH, thyroid-stimulating hormone; LVEDD, left ventricular end-diastolic diameter; LVEF, left ventricular ejection fraction; CAD, coronary atherosclerosis disease; ACE-I, angiotensin-converting enzyme-inhibitor; ARB, angiotensin receptor blocker; MCS, mechanic circulatory support.

### Thyroid Function Tests

The serum concentrations of TSH and thyroid hormones are summarized in **Table 1**. fT3 and TT3 mediumly correlated (r=0.751; *P* < 0.0001) and all results acquired with fT3 were confirmed by TT3 values (<1.28nmol/liter for low fT3 group and ≥1.28nmol/liter for normal fT3 group, respectively). fT3 tended to be mildly but significantly decreased with age (r=-0.357; *P* < 0.0001).

### Low T3 Level and MACE

After a median follow-up of 3.5 ± 2.8 years, cumulative deaths were 9 in low fT3 group and 0 in normal fT3 group (16.7% versus 0, *P* < 0.0001). Cumulative MACE was 16 (9 deaths, 1 heart transplantation, 2 sustained ventricular arrhythmias and 4 re-hospitalizations for heart failure) in low fT3 group and 4 (0 death, 0 heart transplantation, 4 re-hospitalizations for heart failure) in normal fT3 group (29.6% versus 3.5%, *P* < 0.0001). The results of univariate Cox analysis for cumulative MACE endpoint are shown in Table2. In thyroid profiles, fT3 showed a significant predictive value (hazard ratio 0.360, 95% CI 0.223–0.582, *P* < 0.0001). LVEF<50% (hazard ratio 10.231, 95% CI 3.418–30.624, *P* < 0.0001) was another strong predictor of MACE, followed by WBC at admission and age. In model 1 by multivariate analysis, LVEF<50% (hazard ratio 5.184, 95% CI 1.536–17.489, *P* = 0.008) was the strongest predictor of MACE, followed by fT3 (hazard ratio 0.540, 95% CI 0.316–0.922, *P* = 0.024) as a continuous variable. When in model 2, as a categorical variable, fT3 (hazard ratio 5.074, 95% CI 1.518–16.964, *P* = 0.008) was the most important independent predictor of MACE (**Table 2**). Therefore, even after adjustment for traditional risk predictors including LVEF<50%, QRS>120ms and QTc>440ms, low fT3 was a strong independent predictor for MACE in patients with acute myocarditis.

**Table 2:** Univariate and Multivariate Cox Analysis for MACE.

Variables	Model 1			Model 2		
	HR	95% CI	*P* Value	HR	95% CI	*P* Value
**Univariate regression**						
Age, y	1.031	1.002–1.061	0.036			
WBC at admission	1.149	1.038–1.271	0.007			
fT3, pmol/liter	0.360	0.223–0.582	<0.0001			
fT4, pmol/liter	0.944	0.818–1.090	0.432			
TSH, mIU/liter	0.723	0.505–1.036	0.077			
Male				1.195	0.458–3.116	0.716
QRS>120 ms				1.628	0.588–4.504	0.348
QTc>440 ms				2.044	0.815–5.125	0.128
LVEF<50% at admission				10.231	3.418–30.624	<0.0001
fT3<3.54 pmol/liter				10.195	.394–30.625	<0.0001
**Multivariate regression**						
Age, y	1.006	0.976–1.037	0.698	1.004	0.975–1.035	0.774
WBC at admission	1.038	0.931–1.158	0.504	1.056	0.947–1.177	0.327
QRS>120 ms	1.166	0.397–3.423	0.779	1.475	0.509–4.275	0.474
QTc>440 ms	0.904	0.338–2.422	0.841	0.930	0.354–2.445	0.883
LVEF<50% at admission	5.184	1.536–17.489	0.008	4.756	1.456–15.535	0.010
fT3	0.540	0.316–0.922	0.024			
fT3<3.54pmol/liter				5.074	1.518–16.964	0.008

In univariate Cox analysis, Model 1 was constructed on inclusion of continuous variables and Model 2 was constructed on inclusion of dichotomous variables. Multivariate Cox model selected by a stepwise method with factors that were significant in the univariate analysis and traditional risk predictors for prognosis (QRS >120ms, QTc > 440ms, LVEF < 50%). Model 1 was constructed based on inclusion of fT3 as a continuous variable; model 2 was built based on inclusion of fT3 as a categorical variable. WBC, white blood cell; fT3, free triiodothyronine; fT4, free thyroxine; TSH, thyroid-stimulating hormone; LVEF, left ventricular ventricle ejection fraction.

**Figure 2:**
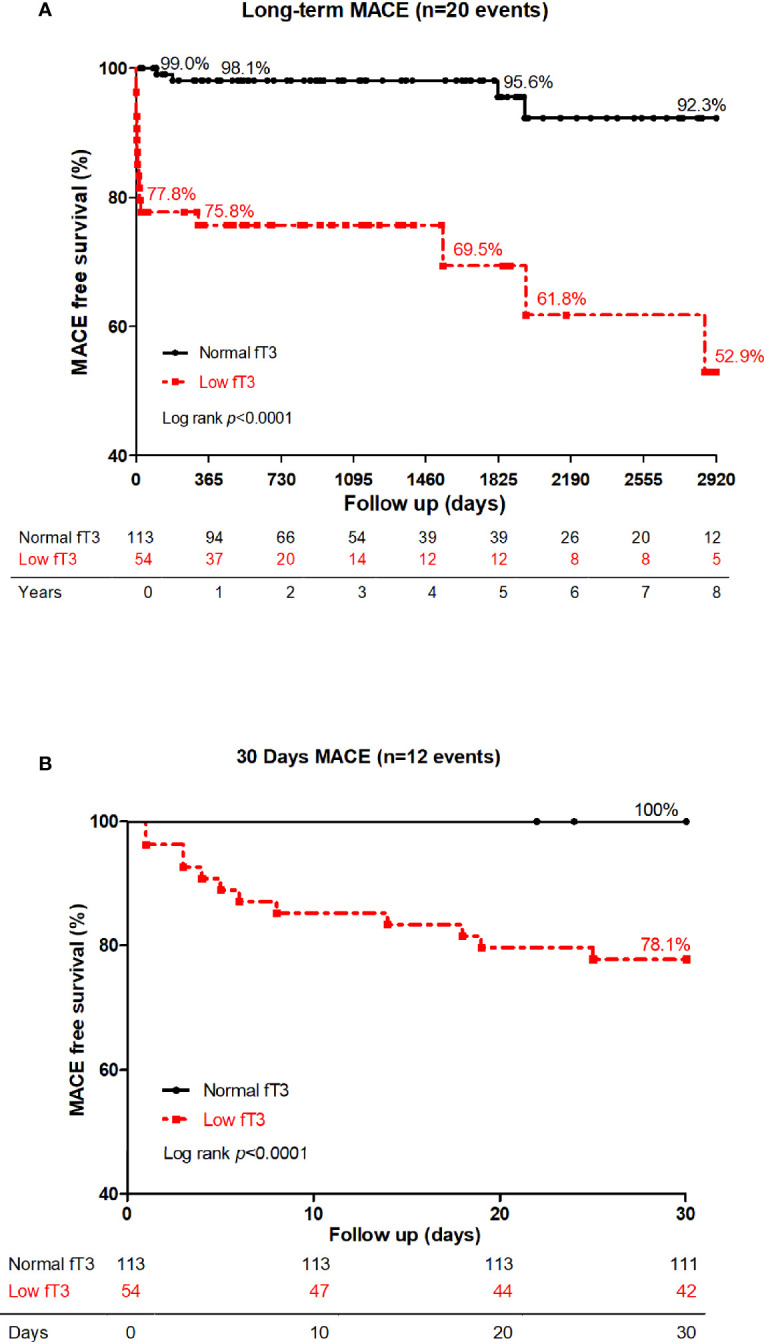
Long-term **(A)** and 30-day **(B)** MACE-free survival in low fT3 group versus normal fT3 group in patients with acute myocarditis. Patients with low fT3 syndrome had more MACE in 30 days as well as in the long-term. MACE included deaths, heart transplantations, re-hospitalization for heart failure and sustained ventricular arrhythmias (>30s).

**Figure 3:**
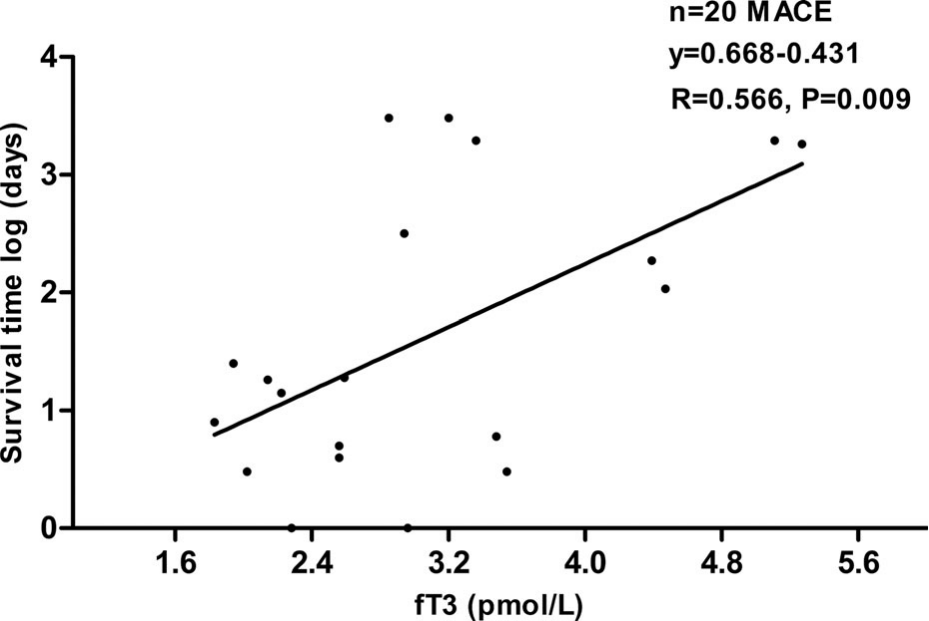
Scatter figure showed a linear correlation between fT3 concentrations and time of survival (days, logarithmic scale) in all patients who had MACE (n = 20). MACE, major adverse cardiac events; fT3, free triiodothyronine.

Long-term and 30-day Kaplan-Meier curves of MACE-free survival in patients with acute myocarditis are shown panel A and B in **Figure 2**, respectively. Difference in MACE-free survival was especially evident in the first month and the survival rate was lower in low fT3 group than normal fT3 group (78.1% versus 100%, log-rank *P* < 0.0001). In the long-term follow-up at 8 years, the MACE-free survival was obviously lower in low fT3 group than normal fT3 group (52.9% versus 92.3%, log-rank *P* < 0.0001). Moreover, a positive correlation was observed between fT3 levels and survival time of the patients who had MACE (**Figure 3**).

**Figure 4:**
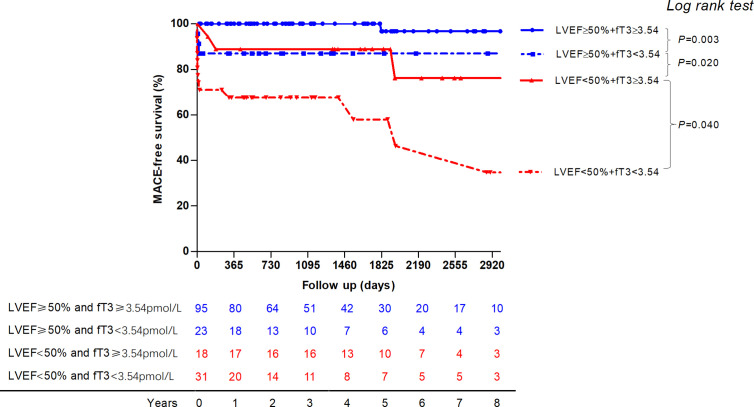
The MACE-free survival probability curve of patients with acute myocarditis and combination of low fT3 status and LVEF are displayed. Patients with LVEF ≥50% and fT3 ≥3.54pmol/liter have a significantly better prognosis compared to those with LVEF ≥50% and fT3 <3.54pmol/liter. Patients with LVEF <50% have worse outcome compared to those with LVEF ≥50%. Patients with LVEF <50% and fT3<3.54pmol/liter have the worst outcome than those with normal fT3 status. MACE, major adverse cardiac events; fT3, free triiodothyronine; LVEF, left ventricular ventricle ejection fraction.

The MACE-free survival probability curves of patients with acute myocarditis and combination of low fT3 status and LVEF are shown in **Figure 4**. Patients with LVEF≥50% and fT3≥3.54 pmol/liter have a significantly better prognosis compared to those with LVEF≥50% and fT3<3.54 pmol/liter (log-rank *P* = 0.003). Patients with LVEF <50% have worse outcome compared to those with LVEF≥50%, with or without fT3≥3.54 pmol/liter (log-rank *P* < 0.0001 and *P* = 0.031, respectively). Patients with LVEF <50% and fT3<3.54 pmol/liter have the worst outcome than those with fT3≥3.54 pmol/liter (log-rank *P* < 0.0001 and *P* = 0.04, respectively).

### ROC Curve Analysis and Predictive Value for MACE

**Figure 5:**
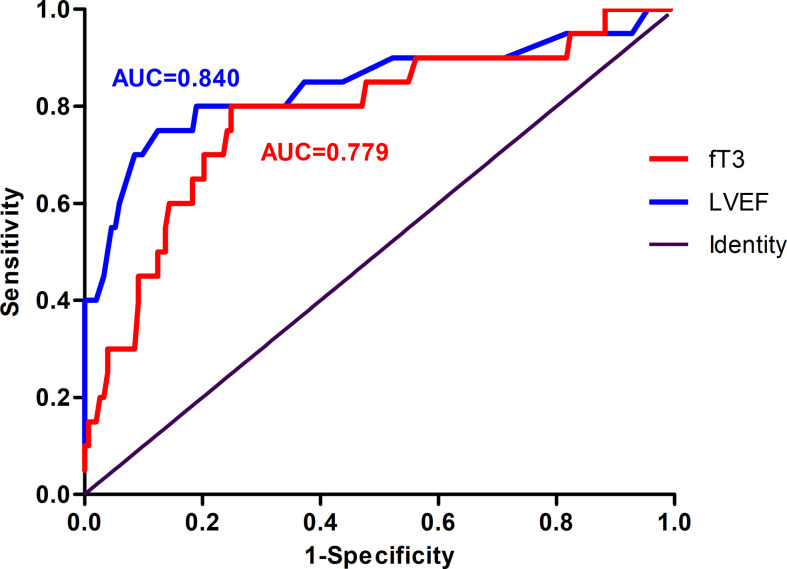
Receiver operating characteristic (ROC) curve of the ability of LVEF and fT3 to predict MACE in patients with acute myocarditis. The area under curve (AUC) for LVEF was 0.840. The sensitivity and specificity were 75.00% and 87.07%, respectively. The AUC for fT3 was 0.779, with 80.00% sensitivity and 74.15% specificity. MACE, major adverse cardiac events; fT3, free triiodothyronine; LVEF, left ventricular ventricle ejection fraction.

To evaluate the predictive value of fT3 and to compare with the traditional MACE predictor LVEF, ROC curves for LVEF and fT3 were both constructed. The sensitivity and specificity of fT3 in predicting long-term MACE in patients with acute myocarditis were 80.00% and 74.15%, respectively (AUC=0.779, optimal cut-off value: 3.565 pmol/liter). The sensitivity and specificity of LVEF to predict MACE were 75% and 87.07%, respectively (AUC=0.840, optimal cut-off value: 41%) (**Figure 5**).

## Discussion

The present study identified fT3 as a significant independent predictor of adverse cardiac outcomes in a cohort of patients with acute myocarditis. Between the two groups, fT3<3.54 pmol/liter status was not only associated with unstable hemodynamic conditions with higher rate of ventricular arrhythmias and worse cardiac function, but also significantly related with a higher rate of MACE including death, heart transplantation, sustained ventricular arrythmia and decompensated heart failure. In multivariate Cox survival analysis, after adjusted for other established risk factors, low fT3 level was significantly associated with cumulative MACE endpoint. In the present cohort, low fT3 was an independent predictor for adverse cardiac events and provided more information for evaluating the long-term prognosis of acute myocarditis. Although low T3 syndrome is well-known in predicting poor prognosis in severe illnesses, this is the first clinical study with a long-term follow-up assessing the prognostic value of thyroid hormone levels in patients with acute myocarditis.

Low serum T3 concentrations are common findings in many patients with severe acute or chronic illnesses including cardiovascular diseases (14). In the past, low T3 syndrome was considered as a useful adaptation of the body to save energy during illness (15). However, accumulating evidence showed that the scale of the decrease in concentrations of thyroid hormone generally represented the severity of the disease, and low T3 level was proved to be a strong predictor of adverse outcomes in patients with cardiac disease (16–18). Iervasi et al (5). reported that low T3 syndrome was a strong independent predictor of poor prognosis in 573 patients with various cardiac diseases including myocarditis (8%). Our previous study (19) showed that thyroid hormone status correlated with cardiac function in patients with dilated cardiomyopathy. Furthermore, we also revealed that fT3 could also serve as a valuable independent predictor of mortality and cardiac transplantation in patients with hypertrophic obstructive cardiomyopathy in another study (20). A meta-analysis including 41 studies (21) showed that the low T3 syndrome was the most prevalent in patients with heart failure, followed by acute myocardial infarction, and was also associated with increased all-cause mortality, cardiac mortality and MACE. Accumulating evidence showed that low T3 status predicted poor prognosis in patients with heart failure and acute myocardial infarction (6, 17, 18, 22).

The mechanisms of low T3 syndrome in acute illness, such as acute myocarditis, include several aspects. Firstly, severe illness results in a downregulation of the hypothalamic-pituitary-thyroid (HPT) axis both at the hypothalamic and pituitary levels, which leads to a decline in serum thyroid hormone concentrations. In autopsy samples of patients with low T3 syndrome, the gene expression of thyrotropin-releasing hormone (TRH) in the hypothalamic paraventricular nucleus decreased, while TRH mRNA expression showed a positive correlation with antemortem serum T3 (23). Secondly, local thyroid metabolism changed in liver and muscle contribute to the low T3 and high reverse T3 (rT3) level. Evidence showed that critical illness induced specific changes in enzymes related to thyroid hormone metabolism such as deiodinases type 1 (D1),2 (D2) and 3 (D3), thyroid hormone transporters, and thyroid hormone receptors (TRα and TRβ) (4, 14). Animal models of acute inflammation induced by lipopolysaccharide showed an upregulation of D2 mRNA expression in tanycytes in the hypothalamus (24), followed by an increase in local conversion of T4 to biologically active T3, then leading to a decrease in TRH mRNA expression in central nucleus (25). Post-mortem tissue of patients died in ICU showed down-regulated D1 and D3 activity in liver and induced D3 in skeletal muscle (26, 27). The ensemble of these changes in deiodinase activities leads to increased inactivation of T4 into rT3 by D3 and decreased activation of T4 into T3 by D1. Severe myocarditis results in injury of myocardium and impairment of cardiac function, which might lead to heart failure and dysfunction of organs, Thirdly, low T3 syndrome was part of the acute phase response to proinflammatory cytokines. Various cytokines including tumor necrosis factor α (TNFα), interleukin-1(IL-1) and interleukin-6 (IL-6) could affect the expression of many proteins connected with thyroid hormone metabolism (14, 24, 28). Previous study showed interferon-α administration in healthy men induced a decrease in serum T3, possibly mediated in part by an increase in IL-6 (29). Solid evidence supports that the mechanism of acute myocarditis is inflammation induced autoimmune injury (8). However, in the present cohort, levels of proinflammatory cytokines were lacking, so further study exploring the exact mechanisms of thyroid dysfunction in acute myocarditis is warranted. Interestingly, low T3 level induced by increased D3 activity locally in granulocyte could optimize bacterial killing capacity (30). Fourthly, loss of appetite and nutritional intake might contribute to the development of low T3 syndrome, which is a favorable adaptation during acute illness. Additionally, in the present study, there were 11.1% and 18.5% of the patients in low T3 group being supported by intubated ventilator and MCS, respectively. These patients were provided totally or partly by parenteral nutrition with decreased caloric intake, which might be an important explanation for low T3 status.

Whether to treat patients with low T3 syndrome remains to be controversial, though some animal and clinical studies reported encouraging findings. Henderson et al. (31) reported that in a rodent model of myocardial infarction-induced chronic heart failure and low T3, T3 replacement to euthyroid status improved systolic function and tended to improve diastolic function, on basis of standard heart failure treatment. Recently, Pingitore et al. (32) reported that oral administration of low T3 dose was safe and able to improve regional cardiac function in patients with acute myocardial infarction and low T3 syndrome. However, studies using T3/T4 or hypothalamic neuropeptides in critical illness so far had neither largely negative results in terms of clinical benefit, nor unclear benefit in lowering mortality. Therefore, large randomized controlled trials are needed to investigate the effects of replacement treatment in terms of clinical outcome.

Despite the encouraging findings, our study has several limitations. First, endomyocardial biopsy, as a gold standard for diagnosis of myocarditis, was performed only in 13.3% of the patients because of reluctance of patients with mild symptoms or high risk of procedure in dynamically unstable patients. Second, due to the time span of 8 years, some patients dropped out, which might lead to incompletion of follow-up information and imprecise assessment of outcomes. Third, some patients without available thyroid function data were critically ill and died before performing a thyroid hormone test, which might introduce selection bias. Fourth, multiple thyroid tests were only available in only small number of the initially evaluated patients, so the present study could not resolve the ambiguity due to a transient change of thyroid status. Last, we performed a retrospective design and single-center study with only limited number of patients, so the present study should be viewed as preliminary, and the results need to be confirmed by large-scale prospective research in the future. Nevertheless, the strengths of our study include the complete information of thyroid function data, long-term follow-up and the exclusion of drug using that might affect thyroid status.

## Conclusion

In conclusion, we identified low fT3 status as a significant independent predictor of poor prognosis for acute myocarditis. The thyroid function profile can provide valuable and convenient information for risk stratification in patients with acute myocarditis.

## Data Availability Statement

The raw data supporting the conclusions of this article will be made available by the authors, without undue reservation.

## Ethics Statement

The studies involving human participants were reviewed and approved by the ethics committees of Fuwai Hospital. Written informed consent to participate in this study was provided by the participants (both of those under the age of 16 and over 16 years) or their legal guardians/next of kin.

## Author Contributions

YZ designed the study, collected the data, and prepared the manuscript. YZ, WW, KZ, and Y-DT performed the statistics. All authors contributed to the article and approved the submitted version.

## Funding

This study was supported by National Natural Science Foundation of China Grant 81825003, Beijing Municipal Commission of Science and Technology Grant Z181100006318005, Chinese Academy of Medical Sciences Initiative for Innovative Medicine (CAMS-I2M) 2016-I2M-1-009 (all to Y-DT).

## Conflict of Interest

The authors declare that the research was conducted in the absence of any commercial or financial relationships that could be construed as a potential conflict of interest.

## Abbreviations

fT3, free triiodothyronine; LVEF, left ventricular ejection fraction; MACE, major adverse cardiac events; HR, hazard ratio; CI, confidence interval; fT4, free thyroxine; TT3, total triiodothyronine; TT4, total thyroxine; TSH, thyrotropin; ROC, receiver operating characteristic; AUC, area under the ROC curve.

## References

[1] Klein I Danzi S. Thyroid Disease and the Heart. Circulation (2007) 116:1725–35. 10.1161/circulationaha.106.678326 17923583

[2] Jabbar A Pingitore A Pearce SH Zaman A Iervasi G Razvi S. Thyroid hormones and cardiovascular disease. Nat Rev Cardiol (2017) 14(1):39–55. 10.1038/nrcardio.2016.174 27811932

[3] Razvi S Jabbar A Pingitore A Danzi S Biondi B Klein I. Thyroid Hormones and Cardiovascular Function and Diseases. J Am Coll Cardiol (2018) 71(16):1781–96. 10.1016/j.jacc.2018.02.045 29673469

[4] Fliers E Bianco AC Langouche L Boelen A. Endocrine and metabolic considerations in critically ill patients 4: Thyroid function in critically ill patients. Lancet Diabetes Endocrinol (2015) 3(10):816–25. 10.1016/S2213-8587(15)00225-9 PMC4979220 26071885

[5] Iervasi G Pingitore A Landi P Raciti M Ripoli A Scarlattini M. Low-T3 syndrome: a strong prognostic predictor of death in patients with heart disease. Circulation (2003) 107:708–13. 10.1161/01.cir.0000048124.64204.3f 12578873

[6] Kannan L Shaw PA Morley MP Brandimarto J Fang JC Sweitzer NK. Thyroid dysfunction in heart failure and cardiovascular outcomes. Circ Heart Fail (2018) 11:e005266. 10.1161/circheartfailure.118.005266 30562095 PMC6352308

[7] Fung G Luo H Qiu Y Yang D McManus B. Myocarditis. Circ Res (2016) 118(3):496–514. 10.1161/circresaha.115.306573 26846643

[8] Caforio AL Pankuweit S Arbustini E Basso C Gimeno–Blanes J Felix SB. Current state of knowledge on aetiology, diagnosis, management, and therapy of myocarditis: a position statement of the European Society of Cardiology working group on myocardial and pericardial diseases. Eur Heart J (2013) 34:2636–48. 10.1093/eurheartj/eht210 23824828

[9] Ammirati E Cipriani M Moro C Raineri C Pini D Sormani P. Clinical Presentation and Outcome in a Contemporary Cohort of Patients With Acute Myocarditis: Multicenter Lombardy Registry. Circulation (2018) 138(11):1088–99. 10.1161/circulationaha.118.035319 29764898

[10] Ammirati E Cipriani M Lilliu M Sormani P Varrenti M Raineri C. Survival and Left Ventricular Function Changes in Fulminant Versus Nonfulminant Acute Myocarditis. Circulation (2017) 136(6):529–45. 10.1161/circulationaha.117.026386 28576783

[11] Hung Y Lin WH Lin CS Cheng SM Tsai TN Yang SP. The prognostic role of QTc interval in acute myocarditis. Acta Cardiol Sin (2016) 32:223–30. 10.6515/acs20150226a PMC4816921 27122953

[12] Ukena C Mahfoud F Kindermann I Kandolf R Kindermann M Böhm M. Prognostic electrocardiographic parameters in patients with suspected myocarditis. Eur J Heart Fail (2011) 13(4):398–405. 10.1093/eurjhf/hfq229 21239404

[13] Anzini M Merlo M Sabbadini G Barbati G Finocchiaro G Pinamonti B. Long−term evolution and prognostic stratification of biopsy−proven active myocarditis. Circulation (2013) 128(22):2384−2394. 10.1161/circulationaha.113.003092 24084750

[14] Boelen A Kwakkel J Fliers E. Beyond low plasma T3: local thyroid hormone metabolism during inflammation and infection. Endocr Rev (2011) 32(5):670–93. 10.1210/er.2011-0007 21791567

[15] Utiger RD. Altered thyroid function in nonthyroidal illness and surgery: to treat or not to treat? N Engl J Med (1995) 333:1562–3. 10.1056/NEJM199512073332310 7477174

[16] Gerdes AM Iervasi G. Thyroid replacement therapy and heart failure. Circulation (2010) 122:385–93. 10.1161/circulationaha.109.917922 20660814

[17] Sato Y Yoshihisa A Kimishima Y Kiko T Kanno Y Yokokawa T. Low T3 syndrome is associated with high mortality in hospitalized patients with heart failure. J Cardiac Fail (2019) 25:195–203. 10.1016/j.cardfail.2019.01.007 30682427

[18] Rothberger GD Gadhvi S Michelakis N Kumar A Calixte R Shapiro LE. Usefulness of serum triiodothyronine (T3) to predict outcomes in patients hospitalized with acute heart failure. Am J Cardiol (2017) 119:599–603. 10.1016/j.amjcard.2016.10.045 28017303

[19] Wang W Guan H Gerdes AM Iervasi G Yang Y Tang YD. Thyroid status, cardiac function, and mortality in patients with idiopathic dilated cardiomyopathy. J Clin Endocrinol Metab (2015) 100(8):3210–8. 10.1210/jc.2014-4159 26052725

[20] Zhang K Meng X Wang W Zheng J An S Wang S. Prognostic value of free triiodothyronine level in patients with hypertrophic obstructive cardiomyopathy. J Clin Endocrinol Metab (2018) 103(3):1198–205. 10.1210/jc.2017-02386 29304228

[21] Wang B Liu S Li L Yao Q Song R Shao X. Non-thyroidal illness syndrome in patients with cardiovascular diseases: A systematic review and meta-analysis. Int J Cardiol (2017) 226:1–10. 10.1016/j.ijcard.2016.10.039 27776249

[22] Song Y Li J Bian S Qin Z Song Y Jin J. Association between low free triiodothyronine levels and poor prognosis in patients with acute ST-elevation myocardial infarction. BioMed Res Int (2018) 16:9803851. 10.1155/2018/9803851 PMC5926512 29850596

[23] Fliers E Guldenaar SE Wiersinga WM Swaab DF. Decreased hypothalamic thyrotropin-releasing hormone gene expression in patients with nonthyroidal illness. J Clin Endocrinol Metab (1997) 82:4032–6. 10.1210/jcem.82.12.4404 9398708

[24] de Vries EM Kwakkel J Eggels L Kalsbeek A Barrett P Fliers E. NF_B signaling is essential for the lipopolysaccharide-induced increase of type 2 deiodinase in tanycytes. Endocrinology (2014) 155:2000–8. 10.1210/en.2013-2018 24635351

[25] Fliers E Alkemade A Wiersinga WM Swaab DF. Hypothalamic thyroid hormone feedback in health and disease. Prog Brain Res (2006) 153:189–207. 10.1016/S0079-6123(06)53011-0 16876576

[26] Peeters RP van der Geyten S Wouters PJ Darras VM van Toor H Kaptein E. Tissue thyroid hormone levels in critical illness. J Clin Endocrinol Metab (2005) 90(12):6498–507. 10.1210/jc.2005-1013 16174716

[27] Peeters RP Wouters PJ Kaptein E van Toor H Visser TJ Van den Berghe G. Reduced activation and increasedinactivation of thyroid hormone in tissues of critically ill patients. J Clin Endocrinol Metab (2003) 88:3202–11. 10.1210/jc.2002-022013 12843166

[28] Kwakkel J Wiersinga WM Boelen A. Differential involvement of nuclear factor-kappaB and activator protein-1 pathways in the interleukin-1beta-mediated decrease of deiodinase type 1 and thyroid hormone receptor beta1 mRNA. J Endocrinol (2006) 189:37–44. 10.1677/joe.1.06354 16614379

[29] Corssmit EP Heyligenberg R Endert E Sauerwein HP Romijn JA. Acute effects of interferon-alpha administration on thyroid hormone metabolism in healthy men. J Clin Endocrinol Metab (1995) 80(11):3140–4. 10.1210/jcem.80.11.7593416 7593416

[30] Van den Berghe G. Non-thyroidal illness in the ICU: a syndrome with different faces. Thyroid (2014) 24(10):1456–65. 10.1089/thy.2014.0201 PMC4195234 24845024

[31] Henderson KK Danzi S Paul JT Leya G Klein I Samarel AM. Physiological replacement of T3 improves left ventricular function in an animal model of myocardial infarction-induced congestive heart failure. Circ Heart Fail (2009) 2:243–52. 10.1161/circheartfailure.108.810747 19808346

[32] Pingitore A Mastorci F Piaggi P Aquaro GD Molinaro S Ravani M. Usefulness of triiodothyronine replacement therapy in patients with ST elevation myocardial infarction and borderline/reduced triiodothyronine levels (from the THIRST Study). Am J Cardiol (2019) 123:905–12. 10.1016/j.amjcard.2018.12.020 30638544

